# Prevalence of *Taenia solium* cysticercosis in pigs entering the food chain in western Kenya

**DOI:** 10.1007/s11250-015-0949-6

**Published:** 2015-11-18

**Authors:** Lian Francesca Thomas, Leslie Jayne Stevenson Harrison, Philip Toye, William Anson de Glanville, Elizabeth Anne Jesse Cook, Claire Njeri Wamae, Eric Maurice Fèvre

**Affiliations:** Centre for Immunity, Infection and Evolution, Institute for Immunology and Infection Research, School of Biological Sciences, Ashworth Labs, University of Edinburgh, West Mains Rd, Edinburgh, EH9 3JT UK; International Livestock Research Institute, PO Box 30709, Nairobi, 00100 Kenya; Royal (Dick) School of Veterinary Medicine, University of Edinburgh, Easter Bush, Edinburgh, Scotland UK; Centre for Microbiology Research, Kenya Medical Research Institute, Nairobi, Kenya; School of Health Sciences, Mount Kenya University, Thika, Kenya; Institute of Infection and Global Health, University of Liverpool, Leahurst Campus, Neston, CH64 7TE UK

**Keywords:** *Taenia solium*, Cysticercosis, Zoonotic, Epidemiology, Public health, Kenya

## Abstract

Three hundred forty-three pigs slaughtered and marketed in western Kenya were subjected to lingual examination and HP10 Ag-ELISA for the serological detection of *Taenia solium* antigen. When estimates were adjusted for the sensitivity and specificity of the diagnostic assays, prevalence of *T. solium* cysticercosis estimated by lingual exam and HP10 Ag-ELISA was between 34.4 % (95 % confidence interval (CI) 19.4–49.4 %) and 37.6 % (95 % CI 29.3–45.9 %), respectively. All pigs, however, were reported to have passed routine meat inspection. Since *T. solium* poses a serious threat to public health, these results, if confirmed, indicate that the introduction of control strategies may be appropriate to ensure the safety of pork production in this region.

## Introduction

Cysticercosis, infection with the intermediate stage of the tapeworm *Taenia solium*, is a zoonotic disease of public health and economic importance and is widespread in Africa, Asia and Latin America (Coral-Almeida et al. 2015). It is a leading cause of acquired epilepsy in humans and a priority for control under the 2012 road map for control of neglected tropical diseases ‘NTD roadmap’ (World Health Organization 2012).

The epidemiological picture for this parasite in sub-Saharan Africa (SSA) is becoming clearer, and it appears that, apart from predominately non-pork-eating areas, *T. solium* is present in practically all countries in the region (Assana et al. 2013). If validated control programmes are to be rolled out as required by the NTD roadmap (World Health Organization 2012), epidemiological data are required to identify areas both in need of control programmes and as baseline data prior to the roll-out of programmes.

Pig keeping in Kenya generally occurs as a small-holder industry with between 2 and 10 pigs per farm with many pigs kept under a free-range system (Kagira et al. 2010), under which they roam over large distances scavenging for food (Thomas et al. 2013). The pigs raised in this way are sold into the local market, either directly to butchers or via traders who travel from farm to farm purchasing pigs (Kagira et al. 2009). Official inspection of these pigs is required by law at the slaughter facility under the Meat Control Act (Government of Kenya 1977). The ad hoc arrangement of the market operating in a largely informal value chain, in conjunction with an understaffed meat inspectorate, however, allows a proportion of pork to enter the food chain without inspection (Kagira et al. 2009). This puts the population at risk from various food-borne diseases, including the parasite *T. solium*. This study investigated the prevalence of *T. solium* cysticercosis in pigs entering the food chain in western Kenya as a first step to understanding the burden of this parasite in this region.

## Method and materials

**Fig. 1:**
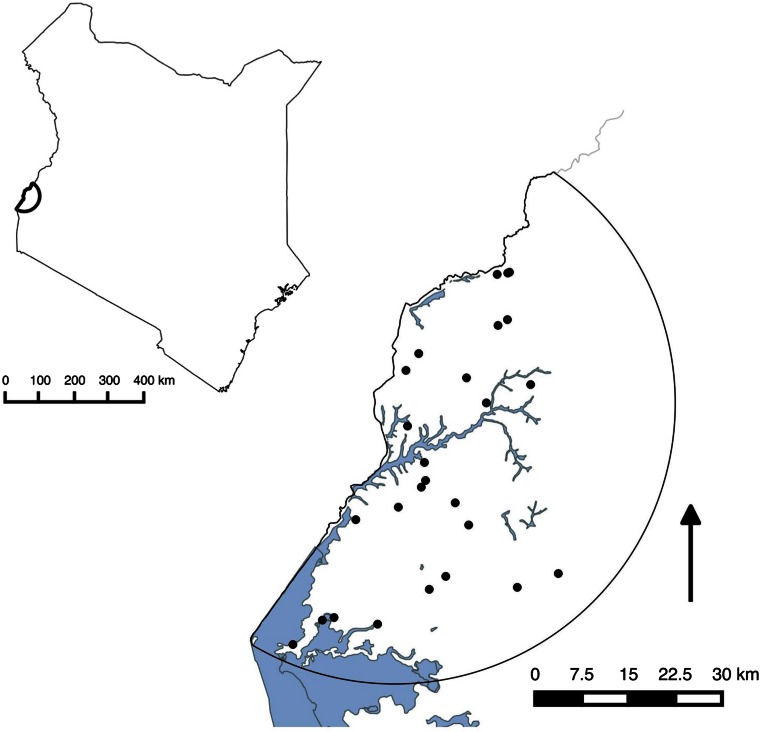
Map of Kenya indicating the location of the study site. The enlargement of the study site depicts the location of slaughter facilities (*black dots*), and the *black arrow* indicates north

The study site, shown in Fig. 1, comprised the 10 divisions with the highest pig populations within a 45-km circumference of Busia town in what was previously western Kenya (now comprising Busia and parts of Siaya Counties). The study area is described in greater detail by Thomas et al. (2013).

A total of 343 sera samples from pigs were collected from porcine slaughter facilities, registered with the district veterinary office, between February and August 2010. A minimum sample size of 319 pigs was calculated to be required, using WinEpiscope 2.0 (Thrusfield et al. 2001) and based upon a maximum assumed *T. solium* prevalence of 14 % (Githigia et al. 2007) with 5 % precision and 99 % confidence level. Slabs were visited on the day of the highest throughput as identified by the meat inspector or slab owner, and all pigs being slaughtered on the day of visit were sampled. Facilities were revisited until a quota proportional to the percentage of the total pig population found in that division had been filled.

Pigs were restrained with a pig snare behind the canine teeth, and a lingual palpation was carried out using a short stick to open the mouth and a cotton swab to protract the tongue. The ventral surface of the tongue was inspected for the presence of cysticerci (Mutua et al. 2007).

Anterior vena cava blood samples (Muirhead 1981) were collected in BD Vacutainer® 10-ml plain tubes and transported to the laboratory on ice, where they were centrifuged at 3000 rpm for 20 min at room temperature. Sera were then separated into two aliquots in 2-ml labelled cryovials and stored at −40 °C until they were transported on dry ice to the International Livestock Research Institute (ILRI) facility in Nairobi where they were stored at −80 °C for between 2 and 7 months prior to laboratory analysis.

Meat inspection results were obtained by contacting the meat inspector on duty for that slaughter facility at the end of the day and requesting his or her report on all pigs slaughtered. The investigators for this study did not interfere in any way with the process of meat inspection as we wished to obtain a true reflection of the activities carried out. A further communication was made with all the meat inspectors involved at the conclusion of the study during which qualitative data were gathered on carcass condemnation for any reason during the time that this survey was taking place. Inspection of pigs at slaughter should be conducted as per the Kenya Meat Control Act (Government of Kenya 1977) which calls for palpation and incision of the tongue, visual inspection and incision of the heart and visual inspection of all exposed muscles, especially the neck, loin, ham and fleshy part of the diaphragm. A carcass found to be infected with any number of cysticerci is judged to be unfit for human consumption, and the whole carcass is condemned. No cysts were collected in this study for morphologic or PCR confirmation.

Laboratory analysis was carried out at ILRI, Nairobi. The HP10 Ag-ELISA (Harrison et al. 1989), modified as described by Harrison et al. (2005) and Krecek et al. (2008, 2011) was used for the detection of active cysticercosis.

Five known negative and two known positive reference controls were run on every plate in the screen.

Plates were corrected for plate-to-plate and day-to-day variations, using a correction factor determined from the first plate of the screening run as described by Harrison et al. (2005).

Cut-off values were determined using the mean corrected optical density (OD) of all negative controls run during the full screen plus three standard deviations (OD = 0.176). Any serum sample with a corrected OD value over this cut-off was counted as being positive.

Results were entered into Microsoft Access 2007 database and were analysed using the ‘R’ environment for statistical computing (R Development Core Team 2005). In order to take into account the sensitivity (Se) and specificity (Sp) of the diagnostic assay, the ‘epi.prev’ function in epiR (Stevenson et al. 2013) was used to adjust the prevalence estimate. The Se/Sp of the HP10 Antigen ELISA has been estimated to be Se 89.5 % (95 % confidence interval (CI) 82.3–94.2) and Sp 74 % (95 % CI 56.6–87.6) (Porphyre et al. 2015), and lingual palpation was estimated to be Se 16.1 % (95 % CI 5–34 %) and Sp 100 % (97.5 % one-sided CI 90–100 %) (Dorny et al. 2004). No adjustment was made for clustering within the population as although sampling took place at a discrete number of slaughter facilities, all pigs originated from separate farms.

## Results

Three hundred forty-three (343) pigs were sampled. One hundred seventy-one (171) pigs were found to be positive by HP10 Ag-ELISA with only nineteen (19) detected to be positive by lingual palpation (see Table 1). The overall prevalence for the study region, adjusting for the current estimates of diagnostic test Se/Sp, is estimated to be 37.6 % (95 % CI 29.3–45.9 %) by HP10 Ag-ELISA and 34.4 % (95 % CI 19.4–49.4 %) by lingual palpation. Reports obtained from the meat inspectors indicated that no pigs with cysticercosis infections were detected at meat inspection and, in fact, no pig carcass or part thereof had been condemned for any reason (including but not limited to cysticercosis) during the time of the study. It is worth noting that on many occasions, no inspector was observed at the slaughter facility. At these times, pig carcasses were seen to leave the premises without inspection. When inspectors were contacted, we were informed that meat had been inspected at the butchery but we are unable to confirm this.

**Table 1:** Prevalence of *T. solium* Cysticercosis in pigs in Western Kenya as determined by lingual palpation and HP10 Ag-ELISA

Division slaughter	Total pigs	Lingual positive	% Prevalence lingual palpation^a^ (95 % CI)	Adjusted prevalence lingual palpation	HP10 positive	% Prevalence HP10 Ag-ELISA^a^ (95 % CI)	Adjusted prevalence HP10 Ag-ELISA
Amagoro	10	0	0 (0–41.2)		4	40.0 (12.2–73.8)	
Amakura	27	0	0 (0–18.3)		12	44.4 (25.5–64.7)	
Budalangi	19	3	15.8 (3.4–39.6)		15	78.9 (31.9–71.33)	
Butula	24	2	8.3 (1.0–27.0)		12	50.0 (29.1–70.9)	
Chakol	14	0	0 (0–31.9)		8	57.1 (28.9–82.3)	
Funyula	69	4	5.8 (1.6–14.2)		43	62.3 (49.8–73.7)	
Matayos	42	2	4.8 (0.6–16.2)		23	54.8 (38.7–70.2)	
Nambale	105	5	4.8 (1.6–10.8)		43	41.0 (31.5–51.0)	
Ugunja	7	1	14.3 (0.4–57.9)		2	28.6 (3.7–71)	
Ukwala	26	2	7.7 (0.9–25.1)		9	34.6 (17.2–55.7)	
Total	343	19	5.5 (3.4–8.5)	34.4 (19.4–49.4)	171	49.9 (44.4–55.3)	37.6 (29.3–45.9)

^a^Crude prevalence not adjusted for Se/Sp

## Discussion

This study has found a high prevalence of pigs testing positive to the HP10 Ag-ELISA, suggestive of viable *T. solium* cysticercosis infections, which places western Kenya within the prevalence range (4–567 %) detected in other endemic areas within Africa (Assana et al. 2013). Pork consumption in the developing countries has been increasing at an astonishing rate (Delgado 2003), and, in keeping with this trend, pig keeping has become a popular small-holder activity for low-income families in western Kenya (Kagira et al. 2010). Zoonotic infections, such as *T. solium*, however, may be a key limiting factor in the economic viability of small-holder livestock keeping (Grace et al. 2012).

The large difference seen between the ‘crude’ prevalence estimates obtained with lingual palpation and HP10 Ag-ELISA (see Table 1) is a direct result of the relative Se/Sp of the two tests. Lingual palpation is a quick, low-cost diagnostic test which has been reported to be used routinely by farmers in Peru to screen their own animals (Gonzalez et al. 1990). It is highly specific for *T. solium* but has very low sensitivity (Dorny et al. 2004; Krecek et al. 2008, 2011), and although it may have a role as a rapid screening tool for endemic areas, it has limited applicability in prevalence studies. The HP10 Ag-ELISA has a much higher sensitivity, though poorer specificity, than lingual palpation. False positives may be detected, including potential cross reactivity with *Taenia hydatigena*, although the prevalence of this parasite is assumed to be low in the pig population of sub-Saharan Africa (Dorny et al. 2004). Adjusting the crude prevalence figures to take these diagnostic characteristics into account provides us with a more ‘true’ impression of the likely parasite prevalence in this region.

The 95 % confidence intervals surrounding these adjusted prevalence estimates are wide and indicate the uncertainty still surrounding the performance of both diagnostic assays for *T. solium* (Porphyre et al. 2015). Here, the estimates of Se/Sp used were sourced from studies utilising non-gold-standard techniques (Dorny et al. 2004; Krecek et al. 2008, 2011; Porphyre et al. 2015). Further work is planned to further explore diagnostic test performance using large-scale fine dissection of carcasses and cyst enumeration as a ‘gold-standard’ diagnostic.

That the pigs sampled in this study were entering the food chain, with no reported condemnation of carcasses by meat inspectors, raises important concerns for public health in this region. Consumption of this infected meat can lead to taeniasis, with any one *T. solium* carrier intermittently shedding thousands of eggs with the potential to cause neurocysticercosis in those people who may unwittingly ingest them (Garcia et al. 2003). A cornerstone of cysticercosis control is preventing infected pork meat from entering the food chain, and our data will therefore be valuable in designing interventions to improve food safety in this setting.

Various options are available for the control of this important zoonotic parasite although evidence for their sustained success is thus far lacking (Carabin and Traoré 2014). Improvement of the meat inspection services has been partially responsible for control of the parasite in Europe (Gemmell 1987). Meat inspection has, however, a notoriously poor sensitivity for the detection of cysticerci (Dorny et al. 2004; Boa et al. 2002; Gonzalez et al. 1990), as evidenced by the data presented here. Resource constraints leading to an understaffed meat inspectorate with insufficient powers to enforce legislation, particularly the condemnation of pigs, also seriously limits the success of meat inspection as a form of control (Thomas 2015).

The resource constraints experienced by the meat inspectorate in western Kenya were illustrated by the many occasions on which no inspector was observed at the slaughter facility. A similar situation was observed in another survey within our study area which found that a meat inspector visited only 84 % (95 % CI 81–87 %) of pig slaughter facilities (Cook 2014).

Should a better resourced inspectorate, and strict enforcement of legislation, be achieved, farmers may be encouraged to utilise the informal slaughter and marketing routes that already operate within western Kenya. This is evidenced by the 3/63 pig farmers who reported utilising informal slaughter in a cross-sectional survey carried out within the same study site (Thomas 2014). It is important therefore that whichever strategy is adopted, all actors in the pork value chain, be they farmers, government institutions or consumers, are sufficiently incentivised to participate in control so that infected pigs are not moved from the formal market into clandestine informal value chains.

## Conclusion

This study indicates that *T. solium* may be endemic in the porcine population of western Kenya but does not appear to be detected at meat inspection. This raises a very real public health concern for the communities who consume this pork and places the economic security of the industry at jeopardy. This area of Kenya should be considered for the roll-out of validated control programmes in accordance with the 2012 NTD roadmap.
